# Allergen sensitization stratifies IL-31 production by memory T cells in atopic dermatitis patients

**DOI:** 10.3389/fimmu.2023.1124018

**Published:** 2023-03-13

**Authors:** Lídia Sans-de San Nicolàs, Ignasi Figueras-Nart, Irene García-Jiménez, Montserrat Bonfill-Ortí, Antonio Guilabert, Laia Curto-Barredo, Marta Bertolín-Colilla, Marta Ferran, Esther Serra-Baldrich, Ramon M. Pujol, Luis F. Santamaria-Babí

**Affiliations:** ^1^ Immunologia Translacional, Departament de Biologia Cel•lular, Fisiologia i Immunologia, Facultat de Biologia, Universitat de Barcelona (UB), Parc Científic de Barcelona (PCB), Barcelona, Spain; ^2^ Departament de Dermatologia, Hospital de Bellvitge, Universitat de Barcelona (UB), L’Hospitalet de Llobregat, Spain; ^3^ Departament de Dermatologia, Hospital General de Granollers, Granollers, Spain; ^4^ Departament de Dermatologia, Hospital del Mar, Institut Hospital del Mar d’Investigacions Mèdiques (IMIM), Universitat Autònoma de Barcelona (UAB), Barcelona, Spain; ^5^ Departament de Dermatologia, Hospital de la Santa Creu i Sant Pau, Universitat Autònoma de Barcelona (UAB), Barcelona, Spain

**Keywords:** atopic dermatitis, CCL27, CLA^+^ memory T cells, house dust mite, IgE, IL-31, periostin, pruritus

## Abstract

**Background:**

The role of allergen sensitization in IL-31 production by T cells and specifically in the clinical context of atopic dermatitis (AD) has not been characterized.

**Methods:**

The response to house dust mite (HDM) in purified memory T cells cocultured with epidermal cells from AD patients (n=58) and control subjects (n=11) was evaluated. AD-associated cytokines from culture supernatants, plasma proteins and mRNA expression from cutaneous lesions were assessed and related with the clinical features of the patients.

**Results:**

HDM-induced IL-31 production by memory T cells defined two subsets of AD patients according to the presence or absence of IL-31 response. Patients in the IL-31 producing group showed a more inflammatory profile, and increased HDM-specific (sp) and total IgE levels compared to the IL-31 non-producing group. A correlation between IL-31 production and patient’s pruritus intensity, plasma CCL27 and periostin was detected. When the same patients were analyzed based on sp IgE and total IgE levels, an increased IL-31 *in vitro* response, as well as type 2 markers in plasma and cutaneous lesions, was found in patients with sp IgE levels > 100 kUA/L and total IgE levels > 1000 kU/L. The IL-31 response by memory T cells was restricted to the cutaneous lymphocyte-associated antigen (CLA)^+^ T-cell subset.

**Conclusion:**

IgE sensitization to HDM allows stratifying IL-31 production by memory T cells in AD patients and relating it to particular clinical phenotypes of the disease.

## 1 Introduction

IL-31 is a clinically relevant neuroimmune cytokine involved in epidermal barrier disruption, pruritus, inflammation and tissue remodeling in atopic dermatitis (AD) (1–3). It is enhanced in the sera from AD patients and correlates with disease severity (4).

The major source of IL-31 are CD4^+^ T cells associated to a Th2 phenotype (5–8), but it is also produced by dendritic cells, mast cells, group 2 innate lymphoid cells, basophils, eosinophils or M2 macrophages (9–14). Its receptor, IL-31RA, is expressed by multiple cell types, including skin sensory neurons, keratinocytes and immune cells, activating the JAK/STAT, PI3K/AKT and MAPK/JNK pathways (9, 15, 16).

IL-31 and IL-31RA expression is increased in the AD skin and are part of the “core” signature characterized by the activation of the IL-31/IL-1 signaling pathway (17). The epidermal barrier function is affected by IL-31 modulation of keratinocyte differentiation directly or *via* other mediators including IL-20, IL-24, IL-33 and IL-1α (18–23). IL-31 binding to its receptor in sensory neurons stimulates itch signaling, neuronal branching and increases of cutaneous nerve fiber density (9, 24). A clear improvement of pruritus has been observed after IL-31RA blockade in moderate-to-severe AD patients (25–27). IL-31 initiates and maintains inflammation by promoting the release of proinflammatory cytokines and chemokines by myeloid cells, eosinophils, basophils and keratinocytes (10, 15, 18–20, 28–31).

Aeroallergens such as house dust mite (HDM) are associated to disease severity and total IgE levels in AD (32, 33). The relationship between IL-31 and allergens has been poorly characterized and mainly limited to canine models: HDM-sensitized dogs showed IL-31 expression in CD3^+^ CD4^+^ T cells in the skin lesions and production of IL-31 by Th2-polarized peripheral blood mononuclear cells (PBMC) stimulated with HDM (7, 34). Non-lesional skin from AD patients challenged with HDM induced IL-31 mRNA expression in three out of six HDM allergic patients (21). Increased frequencies of IL-31^+^ CD4^+^ and CD8^+^ T cells in PBMC from HDM-sensitized patients have also been detected (35). Nevertheless, no studies have addressed the role of allergen in IL-31 production by memory T cells and its possible relationship with particular clinical features in AD patients.

In this study, HDM-induced IL-31 by cutaneous lymphocyte-associated antigen (CLA)^+^ memory T cells correlated with patient’s pruritus. Patients with elevated HDM-specific (sp) IgE (> 100 kUA/L) and total IgE levels (> 1000 kU/L) showed an increased IL-31 production by memory T cells compared to patients with lower sp and total IgE levels. These results show for the first time a relationship between allergen-specific T-cell-mediated IL-31 production, clinical status of the patients and the levels of allergen sensitization that can be of help to guide patient’s identification in response to IL-31-directed therapies.

## 2 Materials and methods

### 2.1 Patients

Peripheral blood and two skin biopsies from active lesional areas were collected from 58 consented moderate-to-severe AD patients and 11 consented controls under institutional review board-approved protocols at the Hospital de Bellvitge, Hospital General de Granollers, Hospital del Mar and Hospital de la Santa Creu I Sant Pau (Spain). Exclusion criteria included topical or systemic anti-inflammatories for the last 2 or 4 weeks prior to the study, respectively.

Plasma samples were assessed for total IgE (kU/L) and HDM-specific IgE (response (OD) and kUA/L) by ImmunoCAP (Thermo Fisher Scientific, Waltham, MA, USA). Serum samples were used for lactate dehydrogenase (LDH) measurement (U/L) in a diagnostic laboratory. Patients’ characteristics are summarized in **Table S1**.

### 2.2 Isolation of memory CLA^+/-^ T cells and epidermal cells suspension

Central and effector memory CD45RA^-^ T lymphocytes were purified from PBMC after Ficoll (GE Healthcare, Princeton, NJ, USA) gradient and three consecutive immunomagnetic separations (Miltenyi Biotech, Bergisch Gladbach, Germany) (36). First CD14^+^ and CD19^+^ cells were depleted, then CD16^+^ and CD45RA^+^ lymphocytes were depleted, and finally CD45RA^-^ T cells were divided into CLA^+^ and CLA^-^ T-cell subpopulations using the Miltenyi product ref 130-092-464. Sample purity was >95% of CLA-positivity for the CLA^+^ T-cell subpopulation and <10% of CLA-positivity for the CLA^-^ T-cell subpopulation (36). Terminal differentiated memory T_EMRA_ cells were not studied with this approach because of CD45RA^+^ cells depletion.

Punch skin biopsy samples were incubated in dispase solution (Corning, Corning, NY, USA) overnight at 4°C, and the epidermal sheet was peeled off from the dermis. The epidermis was cut into pieces and incubated with trypsin (Biological Industries, Kibbutz Beit Haemek, Israel) for 15 minutes at 37°C. Then, the epidermal tissue was mechanically disaggregated by pipetting and the cell suspension was transferred to fresh culture media (1:1 volume) (RPMI supplemented with 10% FBS (Thermo Fisher Scientific) and 1% penicillin-streptomycin (Sigma-Aldrich, St. Louis, MO, USA)). Finally, the epidermal cells suspension (Epi) was obtained by means of centrifugation. It contained all cell types at the same proportion present in the epidermis, being keratinocytes the major cell type.

### 2.3 Coculture of CLA^+/-^ memory T cells with epidermal cells and stimulation

The coculture system was performed by seeding 3 x 10^4^ autologous epidermal cells with 5 x 10^4^ circulating CLA^+^ or CLA^-^ memory T cells (CLA^+^/Epi or CLA^-^/Epi, respectively) in a 96-well U-bottom plate (Falcon, Corning, Corning, NY, USA), in the culture media described above. Whole (CD4^+^ and CD8^+^) CLA^+^ memory T cells were used; however, we assume that results obtained are due to CD4^+^CLA^+^ memory T cells, since the majority of CLA^+^ memory T cells are CD4^+^ (80% CD4^+^ and 20% CD8^+^) and IL-31 is mainly produced by CD4^+^CLA^+^ T cells (6, 23). Cocultures were left untreated (M) or activated for 5 days with HDM extract kindly provided by LETI Pharma at 10 µg/mL or 24 hours with staphylococcal enterotoxin B (SEB) (Sigma-Aldrich) at 100 ng/mL final well concentration (37). In cultures containing only T cells or epidermal cells, the mentioned amounts of each cell type were used, and activation with HDM was performed in the same way. Collected supernatants were kept at -20°C.

For blocking assays, HLA-A/B/C (class I) (BioLegend, San Diego, CA, USA), HLA-DR (class II) (BioLegend), CD1a (Bio X Cell, Lebanon, NH, USA), IL-33 (R & D Systems, Minneapolis, Minnesota, USA) neutralizing antibodies, or respective mouse IgG2a isotype control (BioLegend), mouse IgG1 (BioLegend) and goat IgG isotype control (R & D Systems) were added to cocultures at 10 µg/mL (for HLA and CD1a blocking assays) or 1 µg/mL (for IL-33 blocking assay) final concentration.

### 2.4 Transwell culture

5 x 10^4^ purified CLA^+^ or CLA^-^ memory T cells were added to the top chamber of 0.4 µm pore size polycarbonate 96-well HTS Transwell culture insert (Corning) containing 3 x 10^4^ autologous lesional epidermal cells stimulated with HDM at 10 µg/mL final well concentration in the bottom chamber. Supernatants were collected at day 5 of culture.

### 2.5 Cytokine and chemokine quantification

ProcartaPlex immunoassay (Invitrogen, Waltham, MA, USA) was used to measure IL-31, IL-13, IL-4, IL-5, IL-17A, IL-22 and IFN-γ concentrations in the culture supernatants with the MAGPIX instrument (Invitrogen) and analysed with ProcartaPlex Analyst software version 1.0 (Invitrogen). Values below the lower limit of quantification (LLOQ) were treated as zero. Plasma CCL22 levels were quantified with ProcartaPlex.

### 2.6 ELISA

Pre-coated ELISA kits were used for quantification of plasma levels of CCL27, CCL17, CCL18, sIL-2R (Invitrogen) and periostin (AdipoGen Life Sciences, San Diego, CA, USA).

### 2.7 RNA isolation, quantitative real-time PCR and gene-array analysis

Lesional skin biopsy specimens frozen in Tissue-Tek O.C.T. Compound (Sakura Finetek, Alphen aan den Rijn, The Netherlands) at -80°C were used for RNA isolation by using the TRIzol Reagent (Invitrogen).

For qRT-PCR, cDNA was obtained with the High Capacity cDNA Reverse Transcription kit (Applied Biosystems, Waltham, MA, USA) and preamplified with the TaqMan PreAmp Master Mix (2x) (Applied Biosystems). Taqman Gene Expression Master Mix and FAM-labelled probes (**Table S2**) (Applied Biosystems) were used for qRT-PCR with an ABI Prism 7900HT instrument (Applied Biosystems). Data was processed by SDS analysis software version 2.4.1 (Applied Biosystems) and gene expression was calculated by using the Δ - Δ cycle threshold (Ct) method (with the mean cycle threshold value for RPLP0 and the gene of interest for each sample). The equation 1.8e (CtRPLP0 - Ctgene of interest) x 10^4^ was used for normalizing the values (38).

For gene-array analysis, quality control of concentration and integrity of the isolated RNA was performed with the NanoDrop One (Thermo Fisher Scientific) and the Agilent 2100 Bioanalyzer (Agilent Technologies, Santa Clara, CA, USA). PrimeView Human Gene Expression Arrays (Applied Biosystems) were processed at the Functional Genomics Facility of IRB Barcelona (Barcelona, Spain) and raw data (CEL files) were processed with Transcriptome Analysis Console version 4.0 (Applied Biosystems). Raw data were deposited in the Gene Expression Omnibus repository, accession number GSE226073. Genes with a fold change (FCH) of 1.5 or greater and a p value of less than .05 were considered differentially expressed genes (DEG). Pathway enrichment analysis was performed using g:Profiler web server (https://biit.cs.ut.ee/gprofiler/gost) with Gene Ontology (GO) biological process database and a Benjamini-Hochberg FDR of .05 or greater.

### 2.8 Statistical analyses

Data analysis and representation were performed with GraphPad Prism software version 8 (GraphPad Software Corporation, San Diego, CA, USA). Data are represented as the median ± 95% confidence interval (CI). Wilcoxon test was used to compare two conditions within the same group and Mann-Whitney test was used to compare two groups. In tables, sample median (25th-75th percentiles) and Mann-Whitney test were used for continuous variables, raw numbers (percentages) and Fisher’s exact test were used for categorical variables, and bold values indicate significant data. Correlations were examined using Spearman coefficient and represented with linear regression. Differences were considered significant at a P-value of less than .05 and showed as: (*) p <.05; (**) p <.01; (***) p <.001; (****) p <.0001.

## 3 Results

### 3.1 HDM induces IL-31 in memory T cells cultured with autologous lesional epidermal atopic dermatitis cells, which correlates with pruritus, and plasma CCL27 and periostin

**Figure 1:**
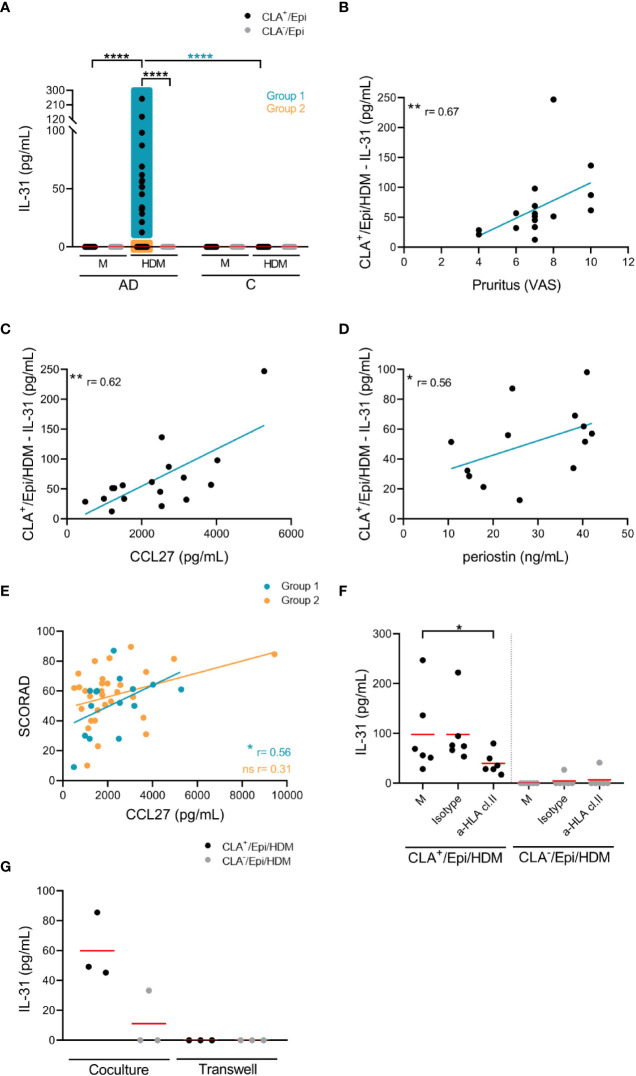
HDM-induced IL-31 by memory T cells correlates with patient’s pruritus and plasma CCL27 and periostin, partially depends on HLA class II molecules and the cell-cell contact with epidermal cells, and is restricted to CLA^+^ T-cells subset. **(A)** IL-31 (pg/mL) produced by CLA^+/-^ T-cell cocultures in basal conditions (M) or stimulated with HDM in AD-(n=58) and C-(n=11) derived samples. Correlations of CLA^+^/Epi/HDM IL-31 production in group 1 with **(B)** pruritus (n=17), and plasma **(C)** CCL27 (n=17) and **(D)** periostin (n=13) levels. **(E)** Correlation between plasma CCL27 levels and SCORAD in group 1 (n=15) and group 2 (n=31) of patients. **(F)** HDM-activated CLA^+/-^ T-cell cocultures were treated with HLA class II neutralizing antibody or control IgG isotype at day 0, and IL-31 production (pg/mL) at day 5 was compared with respect to isotype values (n=6). Red bar indicates the mean. **(G)** CLA^+/-^ T cells were added in the upper chamber and HDM-stimulated epidermal cells suspension in the lower chamber of the 96-well transwell plate and IL-31 (pg/mL) was measured at day 5. Data for three representative patients. AD, atopic dermatitis; C, control subjects; CLA, cutaneous lymphocyte-associated antigen T cells; Epi, epidermal cells suspension; HDM, house dust mite; M, untreated; SCORAD, scoring atopic dermatitis. ns: p >.05; *p <.05; **p <.01; ****p <.0001.

AD-derived cocultures containing circulating memory CLA^+^ T cells and autologous lesional epidermal cells (CLA^+^/Epi) in the presence of HDM led to IL-31 production, whereas CLA^-^ T-cell AD cocultures (CLA^-^/Epi) and control (C)-derived cocultures did not produced IL-31 upon stimulation (**Figure 1A**). Epidermal cells suspension promoted HDM-induced CLA^+^ T-cell-derived IL-31 response (**Figure S1A**) and it was produced in a time-dependent manner (**Figure S1B**).

AD patients were classified into two groups to further characterize clinical profiles based on the differential response to HDM: those producing IL-31 by CLA^+^ T cells were arranged into group 1 (n=17), and those with no IL-31 production were arranged into group 2 (n=41) (**Figure 1A**).

Within group 1 of patients, HDM-induced IL-31 response directly correlated with pruritus (r = 0.67, p = .0036; **Figure 1B**) and plasma levels of CCL27 (r = 0.62, p = .0090; **Figure 1C**) and periostin (r = 0.56, p = .050; **Figure 1D**). Correlations within group 2 of patients could not be performed due to undetectable IL-31 production in the cocultures. As shown in **Figure 1E**, there was direct correlation between plasma CCL27 levels and SCORAD in group 1 (r = 0.56, p = .032) but not in group 2 (r = 0.31, p = .091). Additionally, HDM-induced CLA^+^ T-cell IL-31 nearly correlated with SCORAD in group 1 of patients (r = 0.49, p = .066; not shown). There was no correlation between IL-31 *in vitro* response to HDM and plasma levels of HDM-specific (sp) IgE and total IgE, although there was a tendency for the former (r = 0.46, p = .068; **Figure S2**).

The IL-31 production by CLA^+^ memory T cells was blocked by 59% by a neutralizing HLA class II, but not HLA class I, antibody (**Figures 1F**; **S3A**). Blocking of IL-33 and CD1a molecules did not affect IL-31 production (**Figures S3B, C**), but cell-cell contact between CLA^+^ T cells and epidermal cells was necessary for IL-31 production as demonstrated by transwell cultures (**Figure 1G**).

**Figure 2:**
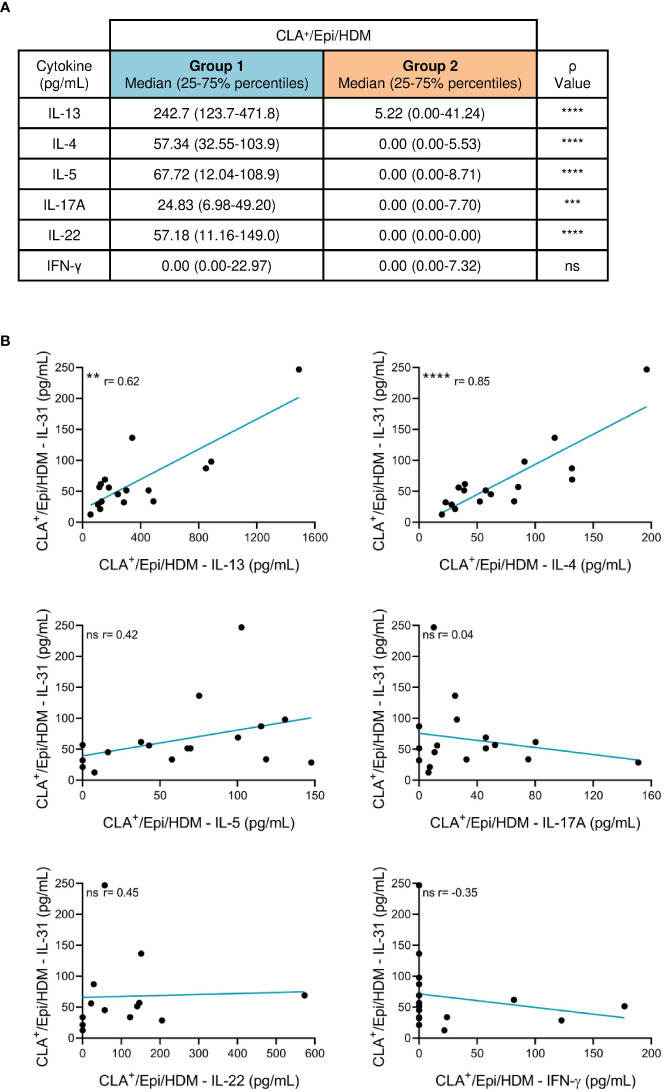
Patients producing IL-31 (group 1) by HDM-stimulated CLA^+^ T cells have a more inflammatory phenotype than patients with undetectable IL-31 (group 2). **(A)** IL-13, IL-4, IL-5, IL-17A, IL-22 and IFN-γ (pg/mL) produced by HDM-induced CLA^+^ T-cell cocultures was compared between groups 1 (n=13-17) and 2 (n=35-41) of patients. **(B)** In group 1, HDM-stimulated CLA^+^ T-cell-dependent IL-31 production was correlated with IL-13 (n=17), IL-4 (n=17), IL-5 (n=17), IL-17A (n=17), IL-22 (n=13) and IFN-γ (n=17). CLA, cutaneous lymphocyte-associated antigen T cells; Epi, epidermal cells suspension; HDM, house dust mite. ns: p >.05; **p <.01; ***p <.001; ****p <.0001.

IL-31 was simultaneously measured with other AD-derived mediators (IL-13, IL-4, IL-5, IL-17A, IL-22 and IFN-γ) and we found that IL-13, IL-4, IL-5, IL-17A and IL-22 production by HDM-induced CLA^+^ T-cell AD cocultures was higher than that by C cocultures (**Figure S4A**). HDM-activated CLA^+^/Epi cocultures from group 1 showed increased production of IL-13, IL-4, IL-5, IL-17A and IL-22 compared to group 2 (**Figures 2A**; **S4B**), revealing a more inflammatory phenotype in group 1 versus group 2. Also, in group 1 IL-31 production directly correlated with IL-13 (r = 0.62, p = .0093) and IL-4 (r = 0.85, p <.0001), but no correlation with IL-5, IL-17A, IL-22 and IFN-γ was observed (**Figure 2B**). Although group 2 of patients were defined for their null production of IL-31 by HDM-stimulated CLA^+^ T cells, there was an heterogeneous IL-13, IL-4, IL-5, IL-17A, IL-22 and IFN-γ response in the CLA^+^ T-cell cocultures of this group (**Figure S5**). All correlations were tested with and without outlier patient data.

When stimulating cocultures with Staphylococcus aureus superantigen B (SEB), another clinically relevant stimulus of AD, IL-31 was specifically produced by CLA+ T cells but no significant differences were found between group 1 and group 2 of patients (**Figure S6A**). Additionally, therewas no correlation between HDM- and SEB-induced IL-31 production by memory T cells (**Figure S6B**).

### 3.2 Patients with memory T-cell IL-31 response to HDM show elevated plasma levels of sp and total IgE and a more inflammatory profile than patients with no IL-31 response

Patients from group 1 displayed increased sp IgE and total IgE levels compared with patients from group 2 (**Table 1**). No differences were found for severity, pruritus, eosinophilia, years since diagnosis and comorbidities. Although group 2 of patients had a reduced allergen sensitization degree when compared to group 1 of patients, they showed higher total IgE and sp IgE levels than controls (total IgE (kU/L): group 2 median = 568.00, C median = 21.10, p <.0001; sp IgE response (OD): group 2 median = 5338, C median = 27.00, p <.0001).

**Table 1:** Differences in clinical characteristics between group 1 and group 2.

Clinical data	Group 1 Median (25-75% percentiles) or Total number (%)	N	Group 2 Median (25-75% percentiles) or Total number (%)	N	ρ Value
Age	28.00 (18.50-44.50)	17	36.00 (27.00-45.75)	40	.063
SCORAD	59.63 (30.00-61.32)	15	59.27 (42.10-68.00)	31	.44
EASI	22.65 (19.38-27.93)	16	25.00 (15.25-32.00)	38	.92
IGA	4.00 (3.00-4.00)	16	3.00 (3.00-4.00)	40	.35
Pruritus (VAS)	7.00 (6.50-8.00)	17	8.00 (6.25-9.00)	40	.31
Eosinophilia (10^3^/uL)	0.73 (0.21-1.06)	16	0.46 (0.24-0.61)	34	.16
**sp IgE response (OD)**	**18747 (12287-20456)**	**17**	**5338 (393.0-16830)**	**41**	**<.0001**
**Total IgE (kU/L)**	**2000 (606.9-5000)**	**17**	**568.0 (110.5-1792)**	**41**	**.0027**
LDH (U/L)	209.0 (143.8-249.8)	6	193.0 (128.0-236.3)	26	.66
Years since AD diagnosis	15.50 (11.00-25.75)	16	24.00 (13.00-33.50)	38	.28
Comorbidities, n	13 (81.25)	16	27 (67.50)	40	.35
Rhinitis, n	8 (50.00)	16	23 (58.97)	39	.56
Asthma, n	6 (37.50)	16	16 (41.03)	39	>.99
Conjunctivitis, n	8 (50.00)	16	15 (38.46)	39	.55
Food allergy, n	2 (12.50)	16	11 (29.73)	37	.30

Categorical variables are presented as counts (percentages) and continuous variables are presented as medians (25th-75th percentiles). AD, atopic dermatitis; EASI, eczema area and severity index; IGA, investigator’s global assessment; LDH, lactate dehydrogenase; OD, optical density; SCORAD, scoring atopic dermatitis; VAS, visual analogue scale. Bold values indicate significant data.

To further characterize both groups, we evaluated changes in lesional skin tissue from patients belonging to group 1 and group 2 using gene-array analysis. We identified two hundred six probe-sets (143 unique genes) up-regulated and one hundred probe-sets (80 gens) down-regulated in group 1 skin compared with group 2 skin (**Figure S7A**; **Table S3**). Among the upregulated genes there were IL-20, IL-24, CCL20, CXCL1, CCL2 and EGR1. Only for IL-20 was FDR of less than .05. Enrichment tests of DEG revealed that group 1 lesional skin was enriched for biological processes related to the immune system such as response to external stimulus, inflammatory response and cellular response to chemokine (**Figure S7B**; **Table S4**). On the contrary, group 2 lesional skin was enriched for developmental processes (**Figure S7C**; **Table S5**).

Interestingly, difference in IL-20 expression between group 1 and group 2 of patients by gene-array analysis was confirmed by qRT-PCR (**Figure S7D**), but higher number of patients should be analyzed to confirm this data due to significant overlap between both groups. Additionally, increase in IL-17A and IL-21 mRNA expression was detected in group 1 compared to group 2 (**Figure S7D**), and no differences were found for IL-31 mRNA expression in cutaneous lesions between both groups.

### 3.3 Patients with sp IgE > 100 kUA/L have increased IL-31 *in vitro* response to HDM confined to the CLA^+^ memory T-cell subset compared to patients with sp IgE < 100 kUA/L

**Figure 3:**
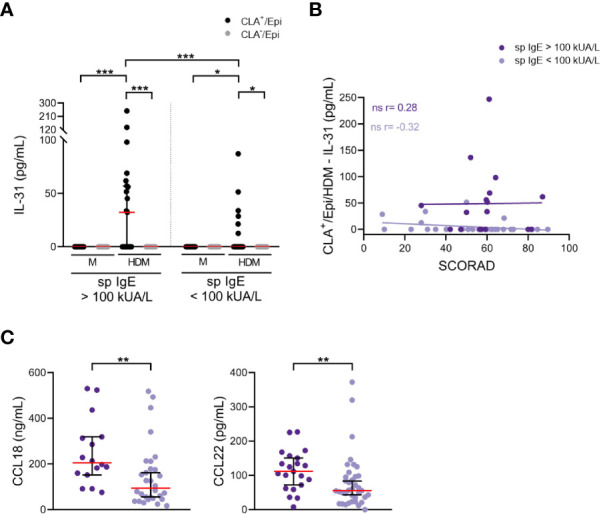
Enhanced IL-31 *in vitro* response and plasma levels of CCL18 and CCL22 in patients with sp IgE > 100 kUA/L. **(A)** IL-31 (pg/mL) from AD culture supernatants separated according to sp IgE levels (> 100 kUA/L n=21, < 100 kUA/L n=37). **(B)** Correlation between CLA^+^/Epi/HDM IL-31 and SCORAD in high (n=17) and low (n=29) sp IgE groups. **(C)** Plasma levels of CCL18 (ng/mL) and CCL22 (pg/mL) were compared between high (n=16-21) and low (n=30-37) sp IgE groups. CLA, cutaneous lymphocyte-associated antigen T cells; Epi, epidermal cells suspension; HDM, house dust mite; M, untreated; sp IgE, specific IgE to house dust mite; SCORAD, scoring atopic dermatitis. ns: p >.05; *p <.05; **p <.01; ***p <.001.

An alternative analysis in the same patients revealed that those with high levels of sp IgE (> 100 kUA/L) showed increased HDM-induced IL-31 response by memory T cells, only in the CLA^+^ T-cell subset, than those with low levels of sp IgE (< 100 kUA/L) (**Figure 3A**). There was a trend towards positive correlation between IL-31 response by HDM-stimulated CLA^+^ T-cell cocultures and SCORAD in the high sp IgE group (r = 0.28, p = .28), whereas this trend was negative for the low sp IgE group (r = -0.32, p = .094; **Figure 3B**). Patients from the high sp IgE group showed increased eosinophilia and total IgE levels compared to patients from the low sp IgE group, as expected (**Table 2**). Additionally, in the high sp IgE there was a tendency towards more patients with conjunctivitis, a sign of HDM clinical symptom (p <.1).

**Table 2:** Differences in clinical characteristics between patients with sp IgE >100 kUA/L and patients with sp IgE <100 kUA/L.

Clinical data	Sp IgE >100 kUA/L Median (25-75% percentiles) or Total number (%)	N	Sp IgE <100 kUA/L Median (25-75% percentiles) or Total number (%)	N	ρ Value
Age	36.00 (23.50-48.00)	21	34.50 (26.25-45.00)	36	.85
SCORAD	59.63 (51.00-62.76)	17	56.00 (37.50-66.58)	29	.52
EASI	27.30 (19.25-34.75)	20	22.10 (11.90-27.41)	34	.13
IGA	4.00 (3.00-4.00)	20	3.00 (3.00-4.00)	36	.21
Pruritus (VAS)	7.00 (6.50-8.00)	21	8.00 (6.25-9.00)	36	.40
**Eosinophilia (10^3^/uL)**	**0.74 (0.47-1.08)**	**19**	**0.33 (0.19-0.56)**	**31**	**.0026**
**sp IgE response (OD)**	**19507 (17643-20456)**	**21**	**4031 (312.5-10513)**	**37**	**<.0001**
**Total IgE (kU/L)**	**3639 (1962-5000)**	**21**	**336.0 (107.0-1130)**	**37**	**<.0001**
LDH (U/L)	237.0 (124.5-329.3)	8	184.5 (122.0-219.3)	24	.13
Years since AD diagnosis	18.00 (13.50-32.75)	20	23.50 (9.50-30.00)	34	.99
Comorbidities, n	17 (80.95)	21	23 (65.71)	35	.36
Rhinitis, n	13 (61.90)	21	18 (52.94)	34	.58
Asthma, n	7 (33.33)	21	15 (44.12)	34	.57
Conjunctivitis, n	12 (57.14)	21	11 (32.35)	34	.094
Food allergy, n	2 (9.52)	21	11 (34.38)	32	.053

Categorical variables are presented as counts (percentages) and continuous variables are presented as medians (25th-75th percentiles). AD, atopic dermatitis; EASI, eczema area and severity index; IGA, investigator’s global assessment; LDH, lactate dehydrogenase; OD, optical density; SCORAD, scoring atopic dermatitis; VAS, visual analogue scale. Bold values indicate significant data.

In line with the previous observation, the IL-31 mRNA expression in cutaneous lesions was greater in the high than in the low sp IgE groups (**Figure S8A**). Furthermore, plasma levels of CCL18, CCL22, CCL27, periostin and sIL-2R were elevated in the high compared to the low sp IgE groups (**Figures 3C**; **S8B**). Although results were significant (p <.05) there was clear overlap between both groups and increased number of patients would be required.

The IL-31 response by memory T cells activated with SEB showed no significant differences between high sp IgE and low sp IgE groups (**Figure S8C**). Additionally, no correlation between the HDM- and SEB-induced IL-31 production was observed in the high sp IgE and the low sp IgE groups (**Figure S8D**).

## 4 Discussion

In moderate-to-severe AD the role of allergen in T-cell mediated IL-31 production and its possible relationship with the clinical status of the patients is presently uncharacterized. Our results suggest that the degree of allergen sensitization allows stratifying patients for IL-31 production by T cells with different clinical features.

In lesional AD skin CD4^+^ T cells are the most abundant infiltrating lymphocytes and they are mainly CLA^+^ T cells (39, 40). Until now, T-cell-derived IL-31 production has been mainly analyzed by qRT-PCR and intracellular flow cytometry of polyclonal-activated PBMC in a low number of patients and without any association with the clinic (8, 21, 23, 35, 41). In our study, HDM-induced IL-31 production by memory T cells defined two groups of AD patients: the IL-31 producing group (group 1) and the IL-31 non-producing group (group 2). In group 1, compared with group 2, HDM-induced IL-31 in CLA^+^ T cells positively correlated with patient´s pruritus intensity, and plasma levels of CCL27 and periostin. In addition, plasma levels of HDM-specific IgE and total IgE were increased in group1.

IL-31 is one of the main drivers of pruritus (2). For the first time, a direct correlation between memory T-cell-derived IL-31 response to HDM *in vitro* and patient’s pruritus was demonstrated, which underlines the clinical relevance of this mechanism in AD. This translational observation stablishes a relationship between allergen and patient’s pruritus through a mechanism restricted to a subset of circulating skin-homing memory T cells. The lack of difference in pruritus intensity between group 1 and group 2 of patients may be due to the broad range of pruritogens in AD, such as IL-13 or IL-4 (1), which could promote the itch signaling pathway in patients that do not produce IL-31 upon HDM stimulation. Since IL-31RA blockade improves symptoms in AD (25, 27, 42), further studies should explore the influence of allergen sensitization and the clinical response to anti-IL-31RA therapies in pruritus.

In group 1 of patients, IL-31 produced by CLA^+^ T cells positively correlates with CCL27, being CLA^+^ T cells and CCL27 mechanistically closely related, since CLA^+^ T cells express the CCR10 receptor that specifically binds CCL27 (43, 44). The relationship between IL-31 producers and plasma CCL27 suggests that lesional keratinocytes produce the CLA-attracting chemokine CCL27 to facilitate further recruitment of CLA^+^ T cells to the inflamed skin where, upon activation with HDM, would produce IL-31. CCL27 in the stratum corneum has been proposed as a biomarker of clinical response to nemolizumab, an anti-IL-31RA, treatment in AD (42), supporting the significant correlation between CCL27 and SCORAD in group 1 of patients. Besides this, not all patients respond to nemolizumab treatment, suggesting that allergen sensitization may be of help to identify anti-IL-31RA responder patients.

Periostin is an extracellular matrix protein closely related to Th2 immune response with an emerging role in pruritus and barrier dysfunction (45–48). In canine models, epicutaneous application of HDM to sensitized dogs induced upregulation of Th2 signature, including IL-31 and periostin (49). Nevertheless, the association between periostin plasma levels and IL-31, for HDM-triggered memory T cells, has not previously been reported.

Group 1 of patients showed increased levels of IL-13, IL-4, IL-5, IL-17A and IL-22, all of them commonly found in AD lesional skin (17), in response to HDM by the CLA^+^ T-cell cocultures, suggesting a strong inflammatory response in this group. Moreover, the direct correlation of IL-31 with IL-13 and IL-4 underlines the allergen-specific and Th2-phenotype of IL-31, and it is also supported by the IL-4-dependent IL-31 production reported in polyclonal stimulated CD4^+^ T-cell clones (5). The skin transcriptome confirmed the inflammatory signature in group 1 of patients, enriched in functions such as “response to external stimulus”, “inflammatory response”, “cellular response to chemokines” and “response to IL-1”. Among the up-regulated genes in this group, IL-20 is associated with IL-31-induced barrier disruption (18), and has also been related with pruritus in murine AD models (50). On the contrary, group 2 of patients lacked a transcriptomic dysregulation of inflammatory genes.

Group 2 of patients responded in most of the endpoints evaluated in this study (*e.g.* severity, pruritus), which is given by the nature that they are AD patients, so they share some clinical characteristics with group 1 of patients. The most significant clinical difference between both groups was the degree of IgE sensitization to HDM, along with total IgE levels, which were related with the differential IL-31 response by skin-derived memory T cells upon stimulation with allergen. Thereby, two groups that apparently would be the same population may show distinct molecular mechanisms driving disease immunopathogenesis.

AD is associated with elevated IgE, sensitization to aeroallergens and eosinophilia (51). Total IgE levels correlate with patients’ severity and are increased in patients sensitized to aeroallergens (33, 52). IgE levels are not a diagnostic requirement, but they are useful for determining prognosis, long-term outcome prediction or choosing therapy (53, 54). When stratifying patients according to their sp IgE levels, the increased IL-31 *in vitro* response in patients with high levels of sp IgE (over 100 kUA/L) supported the HDM contribution to disease activity in AD (32). Noteworthy, the increased IL-31 mRNA expression in lesional skin from high sp IgE patients may explain why topical exposure to HDM induced IL-31 only in a subset of HDM sensitized patients (21). The high sp IgE group also showed enhanced plasma levels of periostin, CCL27, CCL18, CCL22 and sIL-2R, thus associating the allergen-exposure and IL-31 response with Th2-immune response and general inflammation (55). To evaluate signs of HDM clinical symptoms and to compare the reactivity of CLA^+^ memory T cells to HDM with other allergens may be considered to complement these current findings.

A recent research described an increased IL-31RA expression on memory B cells from AD patients with total IgE levels > 1000 kU/L compared to controls, pointing to an association between IL-31-mediated mechanisms and atopic IgE-producing phenotype (56). For this reason, we classified patients according to their total IgE levels into high (> 1000 kU/L) and low (< 1000 kU/L) groups and found an increased HDM-induced memory T-cell IL-31, IL-13 and IL-4 response in the former group (**Figure S9A**), along with enhanced CCL26 mRNA expression in cutaneous lesions (**Figure S9B**) and plasma levels of CCL27, periostin, CCL17, CCL18, CCL22 and sIL-2R (**Figure S9C**). These data suggest that in allergen sensitized patients, IL-31 may play a role not only as T-cell-derived mediator by CLA^+^ T cells but also influencing IL-31RA-expressing B cells. This hypothesis is currently being investigated.

The knowledge generated from the present study is translationally relevant, since the allergen-induced IL-31 production by memory T cells was confined to the CLA^+^ T-cell subset, which reflects the skin-related mechanisms taking place in AD lesions and other inflammatory cutaneous diseases (57–61). Interestingly it has recently been reported that an early-term effect of dupilumab treatment on IL-13, IL-4, IL-5 and IL-22 expression is preferentially found in circulating CD4^+^ CLA^+^ T cells from AD patients (62, 63). Besides this, circulating allergen-specific and CLA^+^ T cells share the same TCRB CDR3 regions as lesional T cells from AD skin (64), supporting the relevance of our results for IL-31 produced by peripheral skin-homing T cells.

Circulating CLA^+^ T cells have been described to be the main memory T-cell subset producing IL-31 (23). When we complemented the analysis of T-cell mediated IL-31 response in the same patients by using SEB (65), IL-31 levels produced by CLA^+^ memory T cells were lower than those observed after HDM stimulation, without significant differences between groups 1 and 2 of patients or sp IgE levels. Additionally, a correlation between HDM- and SEB-induced IL-31 response was not detected, suggesting different roles of these stimuli in T-cell-mediated IL-31 production in AD. The molecular mechanisms by which SEB and HDM activate T cells are different. SEB directly binds to HLA class II molecules on the surface of antigen presenting cells and stimulates T cells expressing particular TCR. SEB can also induce skin inflammation by the following mechanisms: the direct binding to antigen presenting cells in the skin, direct binding to MHC class II keratinocytes, and induction of IgE-mediated immune responses (66). On the other hand, HDM requires uptake, processing and presentation by antigen presenting cells, a process that can be favored by the presence of allergen-specific IgE on the surface of antigen presenting cells (67). Therefore, the IgE-mediated uptake and antigen presentation may be favored in group 1 of patients, since they present higher levels of HDM-specific IgE.

Other inflammatory cells have been shown to produce IL-31: group 2 innate lymphoid cells (ILC2), basophils, eosinophils, dendritic cells, macrophages and mast cells (9–14) A single study has shown IL-31 expression by ILC2 (12), but the clinical relevance of alarmins, which are needed for ILC2 activation, in AD is not clear since all targeted therapies against them failed in clinical trials (68). Regarding basophils, IL-31 has been shown in chronic spontaneous urticaria and healthy donors (10), and no study using AD-derived basophils has shown IL-31 production. In eosinophils, IL-3 induces IL-31 production, but clinical relevance of eosinophils in AD has not been confirmed by anti-IL-5 therapy (14, 68). Dendritic cells express a hundred times less IL-31 than Th2 cells and whether dendritic cells produce physiologically relevant IL-31 quantities is underexplored (9). Recently, a new network comprising IL-31^+^ M2 macrophages, basophils, periostin and TSLP has been described (13); nonetheless, the clinical relevance of these results are unknown due to lack of efficacy of TSLP blockade for AD treatment (68, 69). Mast cells from psoriatic skin and healthy donors express IL-31, but this expression has not been shown for AD (11). Altogether, although IL-31 can be produced by different cell sources besides CLA^+^ memory T cells, no association with clinical pruritus has been reported, therefore their translational relevance is still under investigation. Additional studies on the involvement of IL-31 production by these non-CLA^+^ memory T cells on epithelial cells in coculture assays may be of help to further understand more mechanisms of IL-31 in AD.

This study has some limitations. We tried to detect IL-31 by intracellular flow cytometry but it was a complex issue due to the lack of good tools (5), and plasma or serum IL-31 levels were difficult to detect. Additionally, sample size for gene-array analysis as well as qRT-PCR was limited and more detailed investigations on the interactions between lesional epidermal cells and CLA^+^ memory T cells are needed.

Although the role of allergens in AD is still controversial and at the moment the stratification of response to anti-IL-31RA depending on IgE status has not been performed, it can be hypothesized that assessing specific IgE status to HDM in candidates for anti-IL-31RA therapy may eventually be helpful for identifying patients more prone to good clinical response to anti-IL-31RA and with a more favorable response in comparison to dupilumab or JAK inhibitors.

In summary, our findings bring new data on the mechanisms of allergen sensitization and IL-31 production by memory T cells in moderate-to-severe AD patients. The biphasic IL-31 response to HDM demonstrated with this translational *ex vivo* model may explain the partial IL-31 expression after HDM topical exposure in HDM-sensitized patients (21) and the heterogeneous response to anti-IL-31-directed therapies.

## Data availability statement

The original contributions presented in the study are publicly available in the Gene Expression Omnibus repository, accession number GSE226073.

## Ethics statement

The study involved human participants and was reviewed and approved by the Ethics Committee in Hospital de Bellvitge, Hospital del Granollers, Hospital del Mar and Hospital de la Santa Creu I Sant Pau (Spain). Participants provided written informed consent to participate in this study.

## Author contributions

LS-dSN, IF-N, RP and LS-B contributed to the design of the study. IF-N, MB-O, AG, LC-B, MB-C, MF and ES-B included participants. LS-dSN, IF-N and IG-J participated in the investigation. LS-dSN analyzed the data and prepared figures. LS-dSN and LS-B wrote the manuscript. MF, RP and LS-B contributed to the funding acquisition. RP and LS-B supervised the study. All authors contributed to the article and approved the submitted version.
