# Supplementary figures and images for: Allergen sensitization stratifies IL-31 production by memory T cells in atopic dermatitis patients
Source: Front Immunol. 2023 Mar 13;14:1124018. doi: 10.3389/fimmu.2023.1124018 (PMC10040786; doi:10.3389/fimmu.2023.1124018)

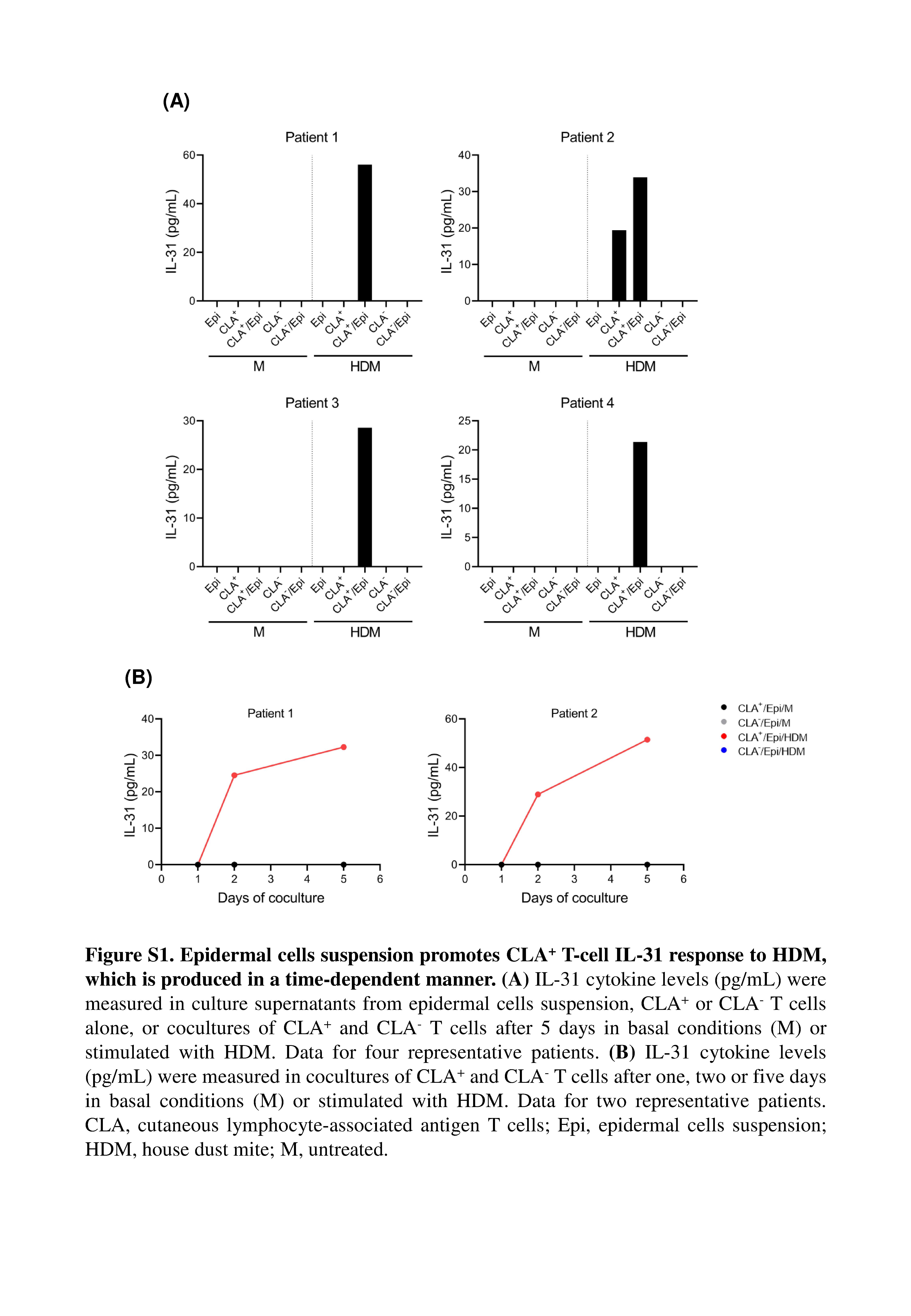

Supplement: Supplementary file 1 [file Image_1.jpg]

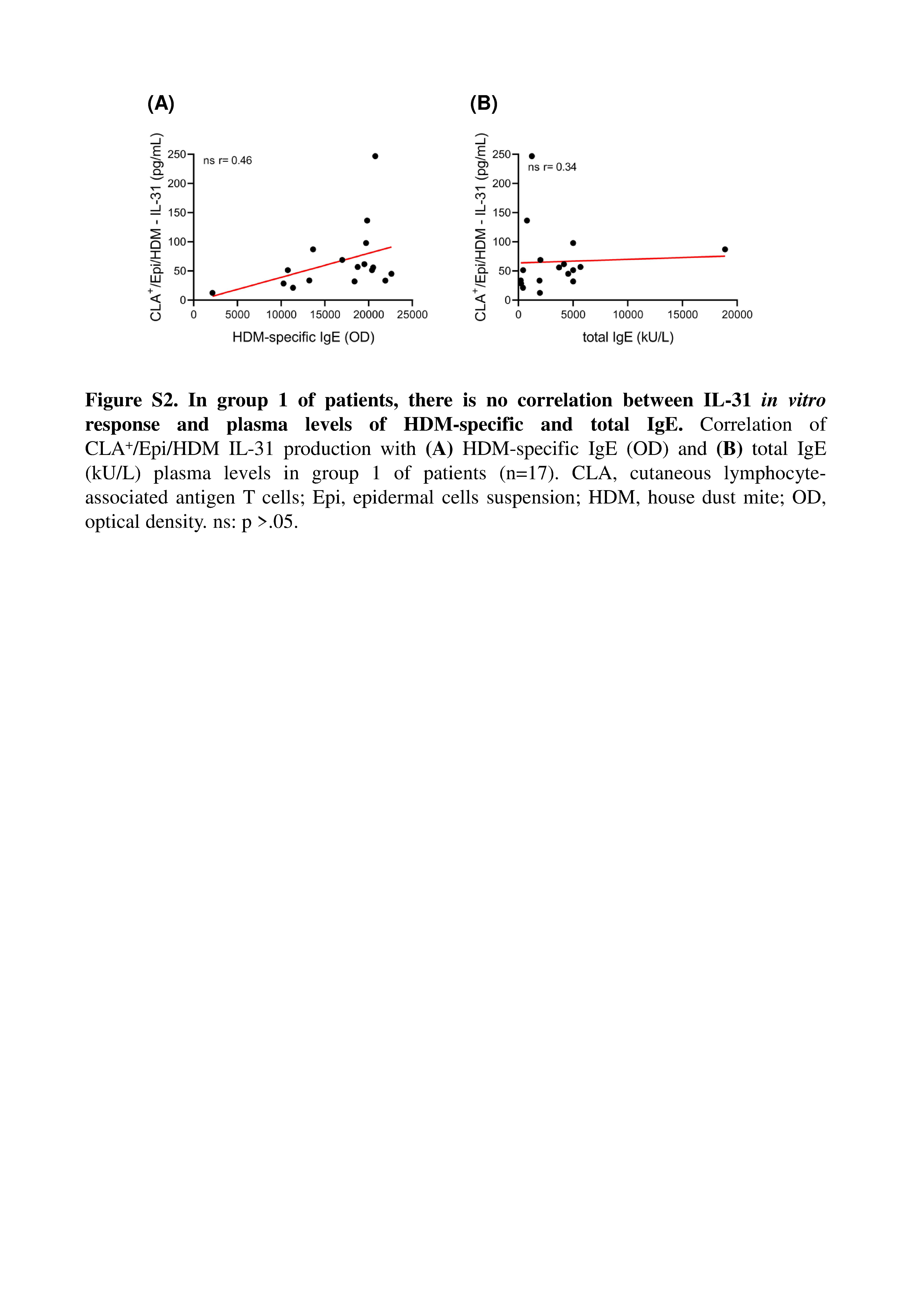

Supplement: Supplementary file 2 [file Image_2.jpg]

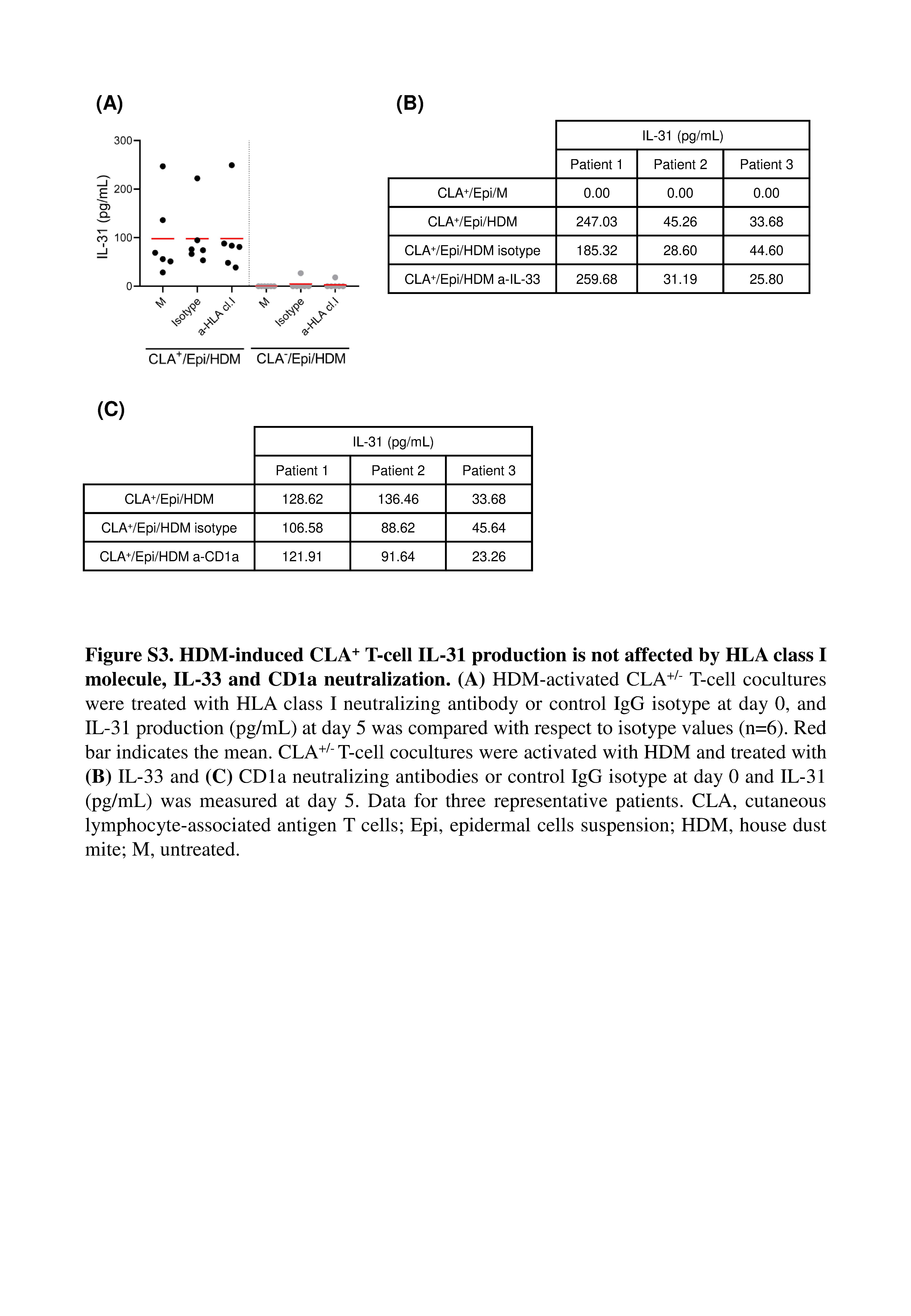

Supplement: Supplementary file 3 [file Image_3.jpg]

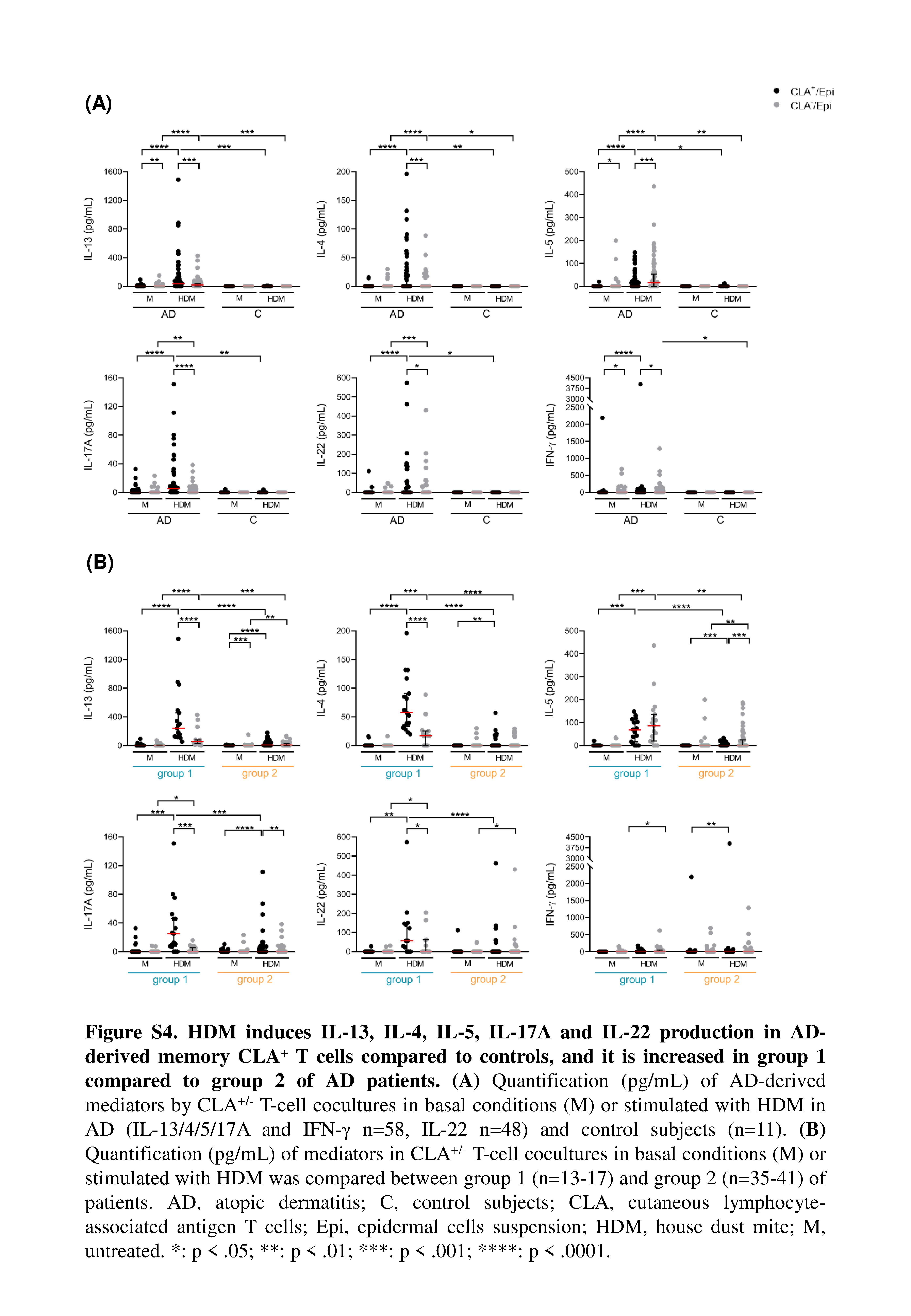

Supplement: Supplementary file 4 [file Image_4.jpg]

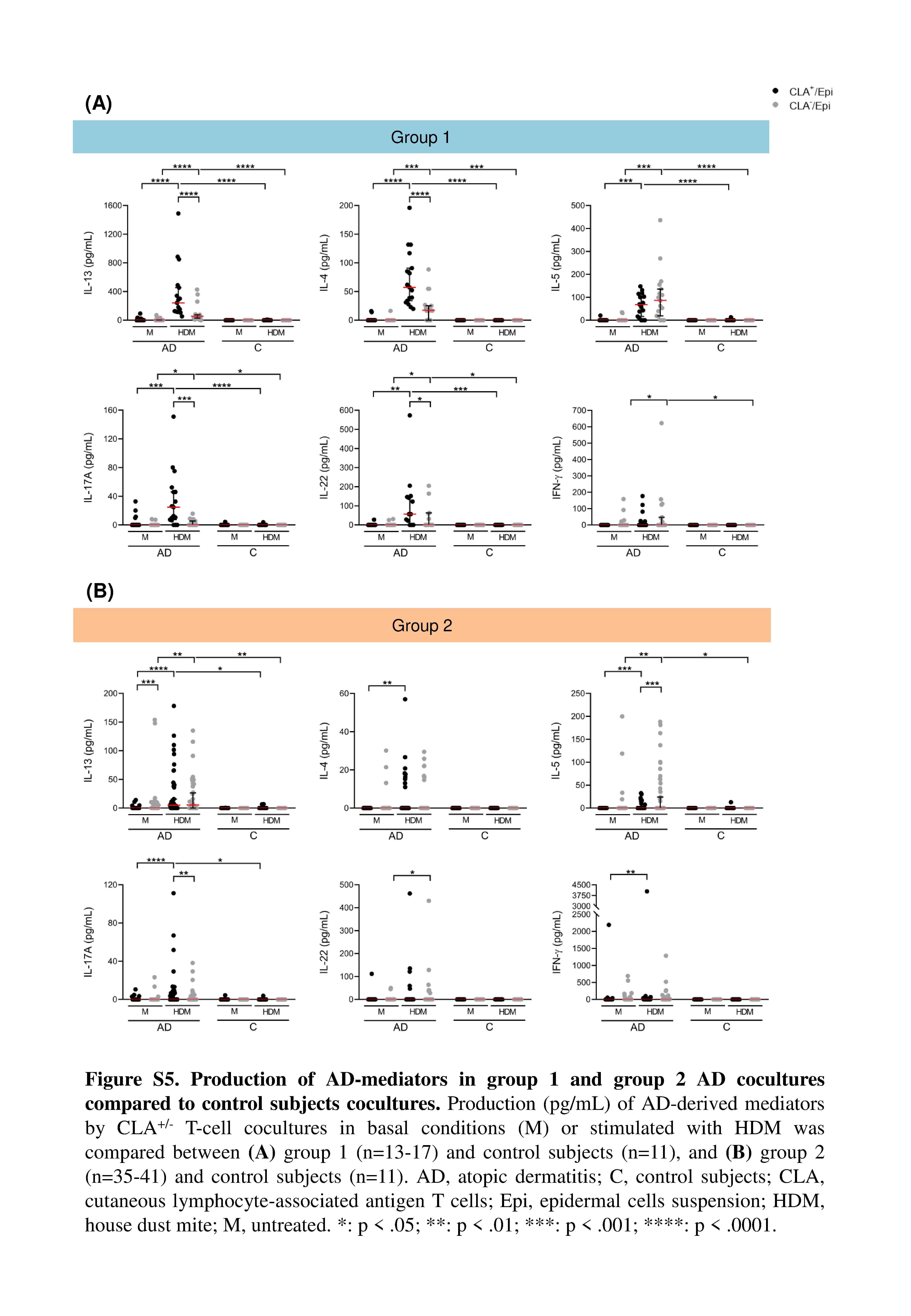

Supplement: Supplementary file 5 [file Image_5.jpg]

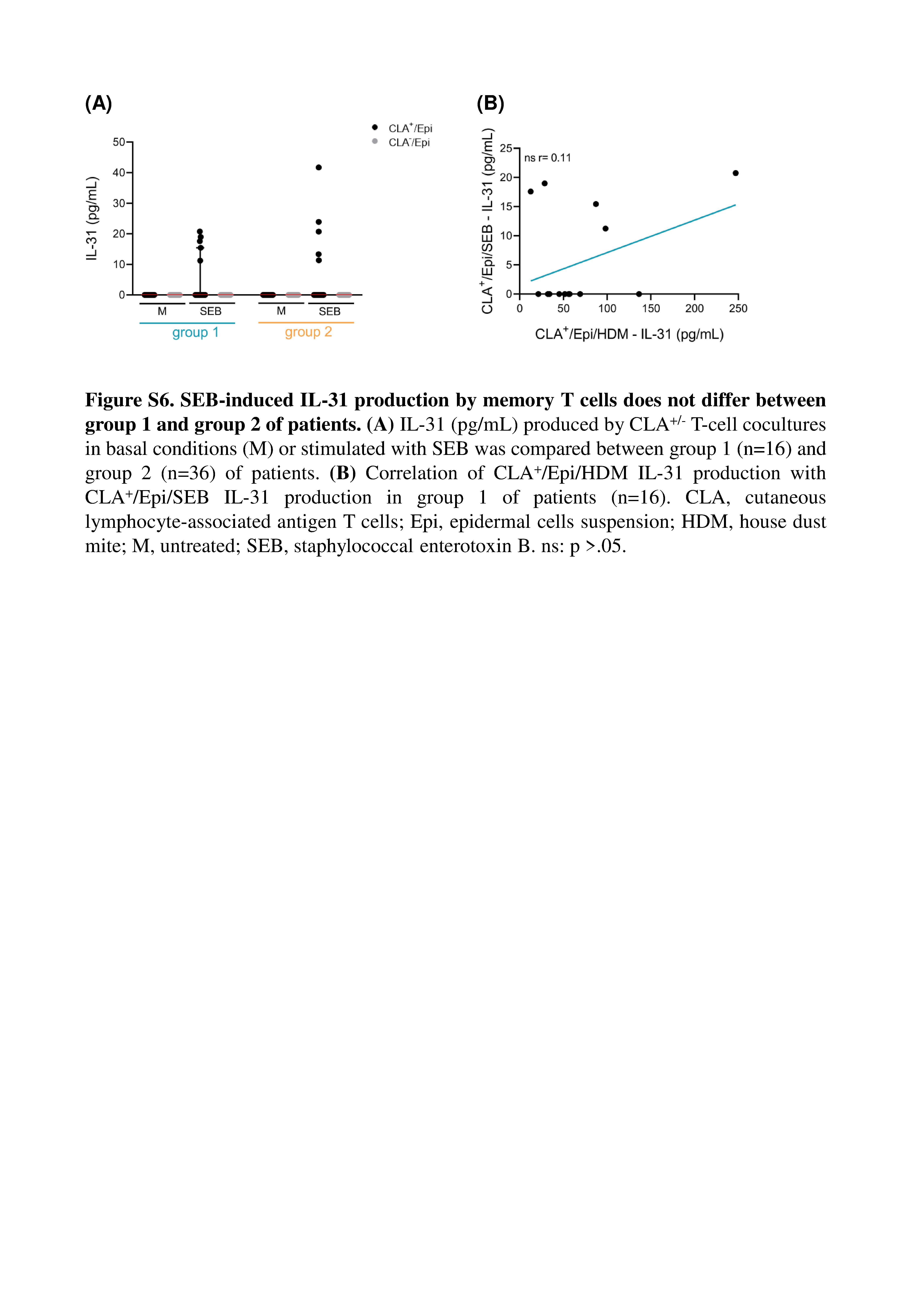

Supplement: Supplementary file 6 [file Image_6.jpg]

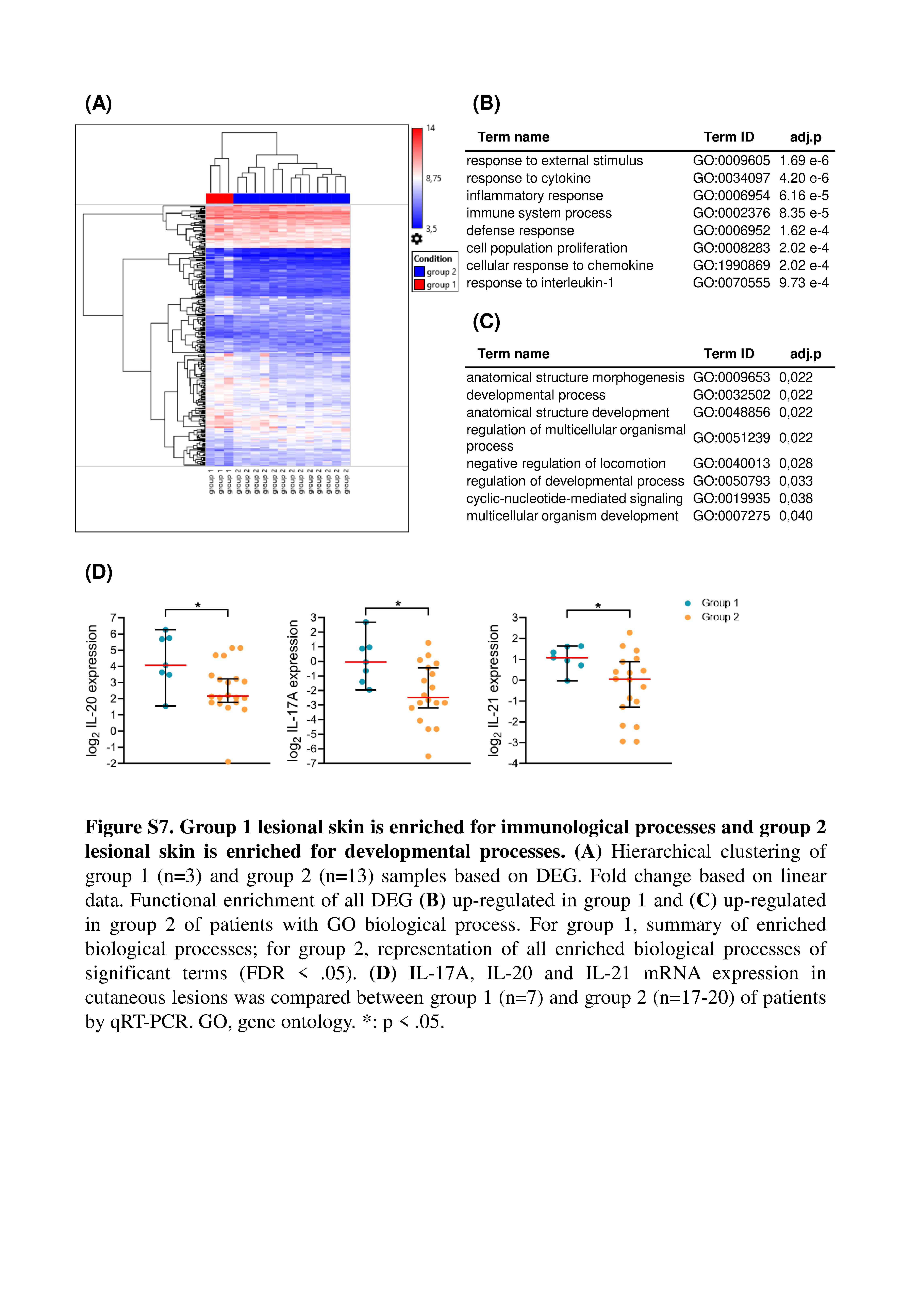

Supplement: Supplementary file 7 [file Image_7.jpg]

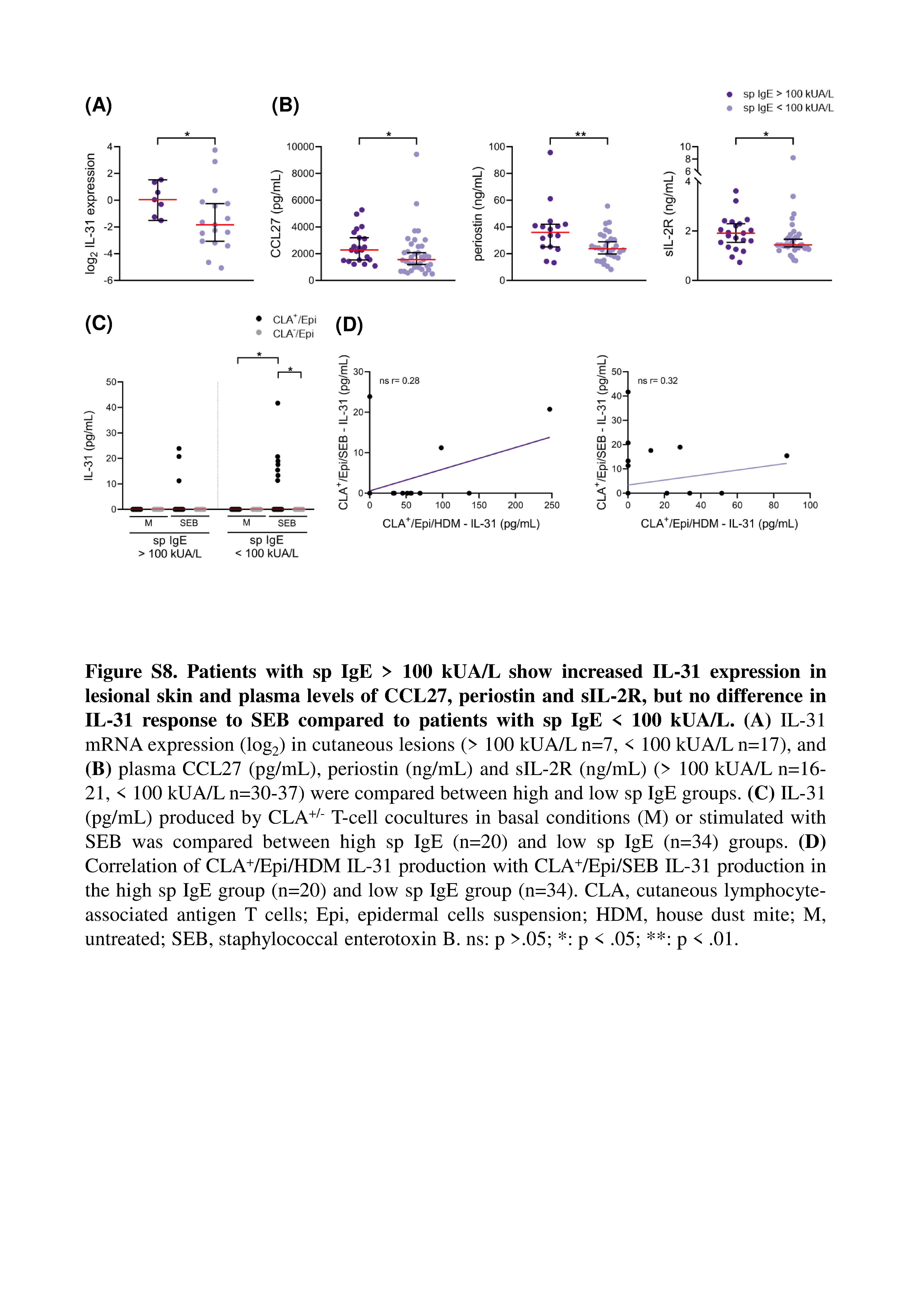

Supplement: Supplementary file 8 [file Image_8.jpg]

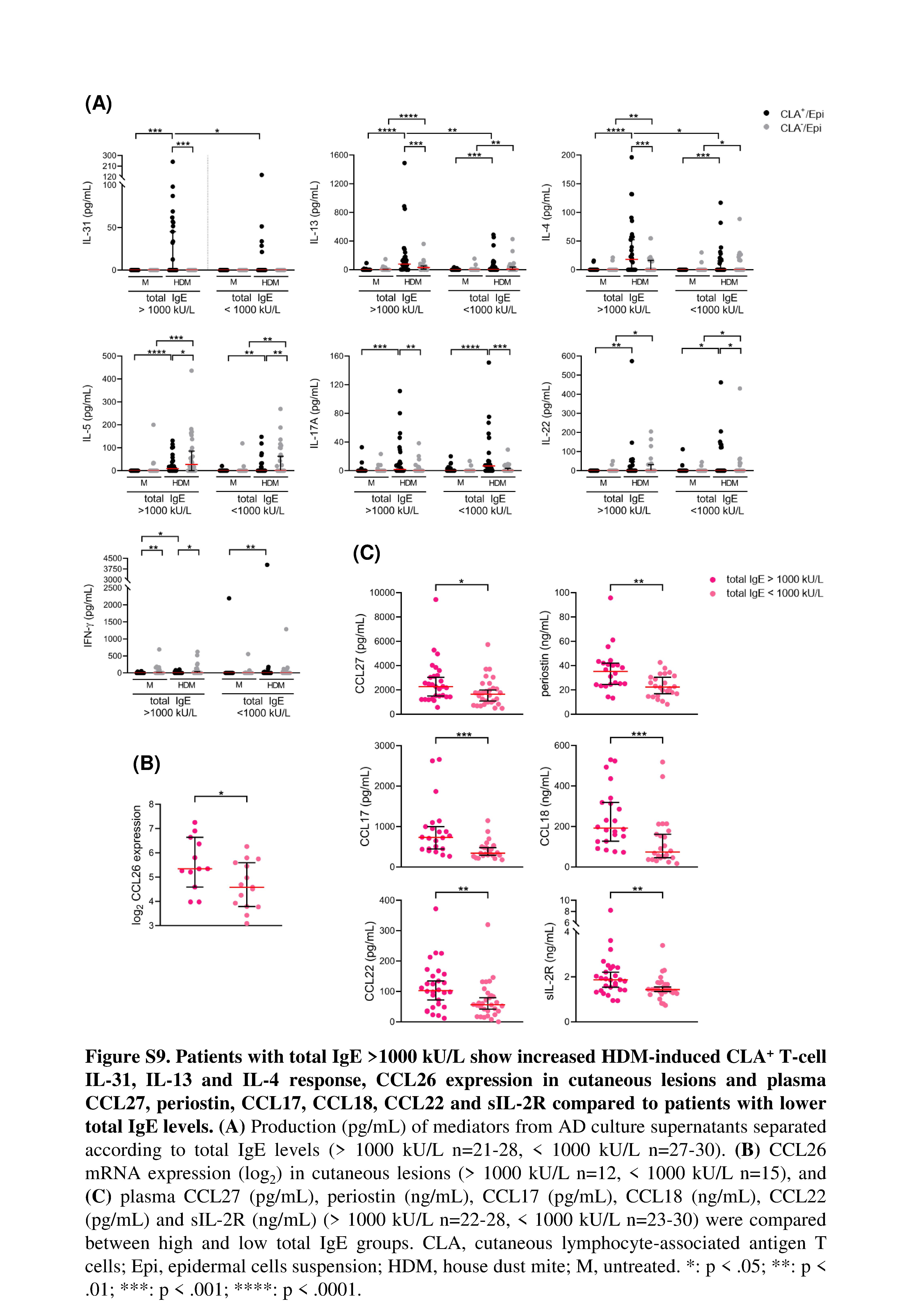

Supplement: Supplementary file 9 [file Image_9.jpg]
